# Responses in honeybee and bumblebee activity to changes in weather conditions

**DOI:** 10.1007/s00442-023-05332-x

**Published:** 2023-02-15

**Authors:** Arrian Karbassioon, Jon Yearlsey, Tara Dirilgen, Simon Hodge, Jane C. Stout, Dara A. Stanley

**Affiliations:** 1 School of Agriculture and Food Science, University College Dublin, Belfield, Dublin, Ireland; 2 UCD Earth Institute, University College Dublin, Dublin, Ireland; 3 School of Biology and Environmental Science, University College Dublin, Dublin, Ireland; 4 School of Natural Sciences, Trinity College Dublin, Dublin, Ireland

**Keywords:** Climate, Humidity, Pollination, Sunlight, Temperature

## Abstract

Insect pollination, and in particular pollination by bees, is a highly valued ecosystem service that ensures plant reproduction and the production of high-quality crops. Bee activity is known to be influenced by the weather, and as the global climate continues to change, the flying frequency and foraging behaviour of bees may also change. To maximise the benefits of pollination in a changing world, we must first understand how current weather conditions influence the activity of different bee species. This is of particular interest in a country such as Ireland where inclement weather conditions are nominally sub-optimal for foraging. We observed honeybee (*Apis mellifera*) and buff-tailed bumblebee (*Bombus terrestris*) activity across a variety of weather conditions at seven apple orchards to determine how four weather variables (temperature, relative humidity, solar radiation, wind) influenced the flight activity of each species. Each orchard contained three honeybee and three bumblebee colonies, and so we were able to observe a colony of each species concurrently in the same weather conditions. Overall, honeybees were more sensitive to changes in weather than bumblebees and could be more predisposed to future changes in within-day weather conditions. Our results indicate bumblebees could compensate for low honeybee activity in inclement conditions, which supports the theory that pollinator diversity provides resilience. This may be particularly important in management of pollinators in crops that flower in the spring when weather is more variable, and to allow varied responses to global climate change.

**Supplementary Information:**

The online version contains supplementary material available at 10.1007/s00442-023-05332-x.

## Introduction

Insect pollinators play a key role in domestic crop production and the maintenance of wild plant communities worldwide (Corbet et al. 1991; Klein et al. 2007; Hung et al. 2018). Rodger et al. (2021) estimate that the seed production of 79% of flowering plant species benefit to some degree from animal-mediated pollination. Pollination services contribute greatly to the world economy, with their contribution to global crop production valued at 235–577 billion US dollars per year (IPBES 2016). The efficacy of a pollinator species in a system can in part be calculated by how many flowers it visits in a given time and how much pollen is attached to and deposited from its body per visit (Ne'eman et al. 2010). Visitation rates are influenced by the weather (Vicens and Bosch 2000); weather can determine the flight speed, flight duration, and foraging behaviour of bees (Wratt 1968; Heinrich and Heinrich 1983; Corbet et al. 1993; Woods et al. 2005; Abou-Shaara 2014). Of the bees, honeybees (*Apis mellifera* L.), and bumblebees (*Bombus* spp*.*), are important crop pollinators in temperate regions around the world (Kleijn et al. 2015).

In agriculture, honeybees (*Apis mellifera*) are primarily managed for their pollination services, where they account for approximately 50% of global crop pollination (Kleijn et al. 2015) as they increase crop yield and nutritional value (De la Rua et al. 2009; Burns and Stanley 2022). Although some bumblebees are managed for pollination (Osterman et al. 2021), most flower visitation is carried out by unmanaged populations. In some cases, bumblebee pollination has been shown to supplement or even surpass that of honeybees (Willmer et al. 1994; Zhang et al. 2015). For example, Pérez-Méndez et al. (2020) found the absence of bumblebees in apple orchards responsible for losses in fruit set and number of fruits per apple tree, despite honeybee visitation.

Honeybees will fly in warm temperatures up to ~ 42 °C (Atmowidjojo et al. 1997) but reduce flights with increases in precipitation, wind speed, humidity, and cloud cover (Nielsen et al. 2017; Lawson and Rands 2019). Bumblebees are less tolerant to high temperatures than honeybees but are not as perturbed by lower temperatures, inclement weather, and low light conditions (Dag et al. 2006; Reber et al. 2015). This suggests that bumblebees will compensate for the relatively lower activity of honeybees in less favourable conditions.

Ireland acts as a buffer for the European continent from Atlantic weather forces, resulting in a unique climate characterised by highly variable weather (Wheeler and Mayes 1997). This variability makes Ireland an ideal location for assessing the effects of weather on bees, and, as Irish weather is distinct from the rest of Europe, a study here could provide valuable insights as to how pollinator behaviour may change under different climate change scenarios (Sanderson et al. 2015). Although the effects of weather on honeybee and bumblebee activity have been previously explored (Corbet et al. 1993; Tuell and Isaacs 2010; Lee et al. 2016), simultaneous observations of the two species at the colony are scant; at the time of writing, the only study on the effect of weather on pollinator activity in Ireland was made by Mahon and Hodge (2022), although this was performed at flowers, not the colony, and only within a narrow, pre-determined range of weather conditions.

The main objectives of this study were to identify how weather, and specifically which weather variables, influence honeybee and bumblebee flight activity and foraging behaviour. To do this, we made concurrent observations of honeybee (*Apis mellifera*) and buff-tailed bumblebee (*Bombus terrestris*) colony entrance activity at seven apple orchards in Ireland across a spectrum of weather conditions, and specifically asked the following questions:

i. How does weather influence the number of honeybees exiting, returning to the colony, and returning to the colony with pollen loads?
ii. How does weather influence the number of bumblebees exiting, returning to the colony, and returning to the colony with pollen loads?

## Materials and methods

### Study system

**Fig. 1:**
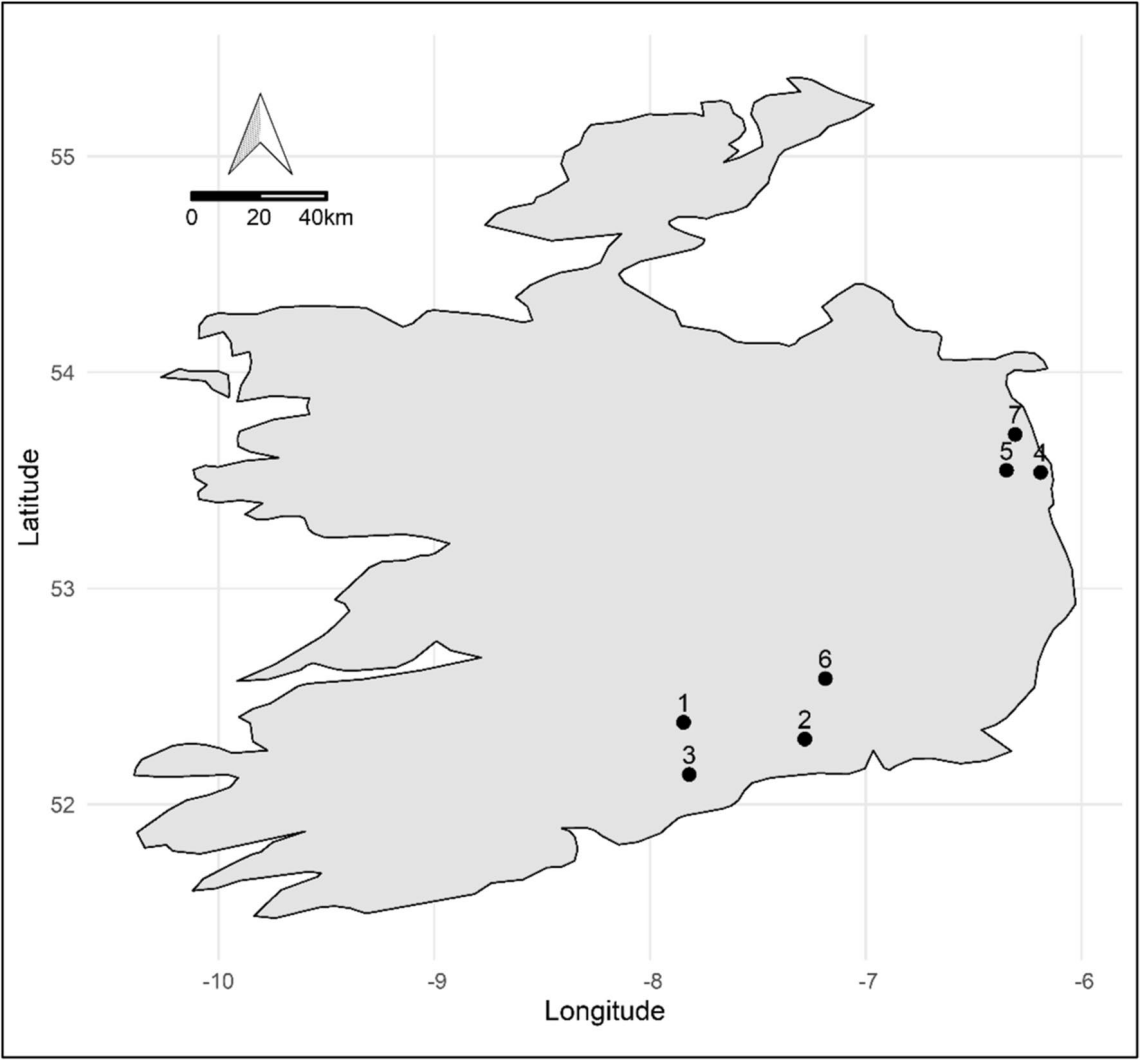
Map of apple orchard sites across the Republic of Ireland. We observed three honeybee and three bumblebee colonies at each site. Sites 4, 5, and 7 are in a region which historically experiences more inclement weather than the region in which 1, 2, 3 and 6 are situated

We conducted observations of bee activity from 26 April to 28 May 2019, during the flowering period of apple. To extend the range of observed weather conditions and the number of replicates for honeybee and bumblebee colonies, we selected seven apple orchards along a geographic range in eastern Ireland, with 202 km between the northernmost and southernmost sites (Fig. 1; Table S1). The northernmost sites are in an area which, on average from 1981 to 2011, was less sunny and experienced more precipitation than the southernmost sites (Met Éireann 2012). The orchards varied in size from 0.5 to 50 hectares and tree densities of 332–3135 trees per hectare.

The honeybee colonies of the native honeybee sub-species *Apis mellifera mellifera* were supplied and maintained by local beekeepers. We obtained research colonies of *Bombus terrestris audax* bumblebees from Biobest via Agralan Ltd., Wiltshire U.K., with colonies containing a queen and approximately 80 workers upon delivery. At each site, we placed three honeybee colonies 1 m apart from each other along hedges facing south/south-east into each orchard, and along the same hedge at least 5 m from the nearest honeybee colony, we placed three bumblebee colonies, also 1 m apart each. Only the honeybee colonies at site 7 had been turned away from the orchard and towards the hedge for visitor safety.

### Activity data gathering

We defined the activity of a colony as the number of bees exiting or returning to the colony, with those returning bees designated as carrying a visible pollen load or not. An observation consisted of all activity recorded for 5 min at the colony entrance, and we made two consecutive observations per colony before moving to the next one. Colonies were always observed from left to right, and we allotted a 10-min interval before moving to the next colony to allow for any changes in the weather over time. Four 5-min observations of each colony were made per site visit, and we visited each site at least six times. To the best of our ability, we observed the sites in the widest range of weather conditions possible, and at an equal number of times in the morning (09:00–12:00) and afternoon (12:00–17:00), as bees have been shown to exhibit bimodal patterns of activity with peaks in the morning and the afternoon (Xu et al. 2021).

We recorded bumblebee activity in the field by eye. The small size of a bumblebee colony (typically 300–400 individuals) and the low rate of activity (1 or 2 individuals passing through at a time) made it feasible to do so, with each observer standing 3–4 m away from the colony so as not to interfere with any flights. While observing bumblebees, we simultaneously recorded honeybee colony returns with a video camera (Canon Legria HFR806), which allowed us to obtain data for both species largely under the same weather conditions.

Videos of honeybees were subsequently watched in the lab and data recorded. Following pilot observations, we found that activity in the third minute of each 5-min video was closely correlated with activity in the full observation, so we multiplied the count from this third minute by five to obtain our recordings of honeybee activity. Any videos of swarming honeybees were removed as they were not representative of typical honeybee behaviour as determined by the weather.

### Weather data recording

We recorded temperature (°C), solar radiation (W m^−2^) and relative humidity (%) at intervals of 30 s using a HOBO Micro Station Logger from Onset Computer Corporation. Temperature was measured to a resolution of 0.02 °C, relative humidity to 0.1%, and solar radiation to 1.25 W m^−2^. We situated the logger equidistantly between the honeybee and bumblebee colonies and levelled the logger sensors between the heights of the honeybee and bumblebee colony returns to capture the microclimate at this height. We also recorded wind on the Beaufort scale prior to each observation.

### Data processing, exploration, and analysis

The average weather conditions for each 5-min observation were calculated using native R programming language (R Development Core Team 2020).

We wanted to determine the influence of each weather variable on each measure of bee activity. Even though temperature, relative humidity, and solar radiation are often inter-related, we retained them all in each model to explore their individual effects (Wratt 1968; Woods et al. 2005; Tuell and Isaacs 2010; Reber et al. 2015; Clarke and Robert 2018). To estimate the inflation in standard error of our model coefficients due to collinearity, we calculated the ratio of squared standard errors for each term’s fitted coefficient, a value comparable to a Variance Inflation Factor. In all cases, we determined the effect of standard error inflation to be low (Table S2). Prior to model fitting, we scaled and centred each weather variable. After plotting the weather variables against each type of activity and detecting unimodal trends, we decided to also include the quadratic terms of the three continuous weather variables in our models.

We fitted generalised linear mixed models to each type of activity—returning without pollen, returning with pollen, and exiting—for both honeybees and bumblebees in R using the package glmmTMB (Brooks et al. 2017). We included covariates of the linear and quadratic terms of temperature, relative humidity, and solar radiation, as well as interactions between the linear terms and the ordinal factor wind. To determine if bee return rate was influenced by pollen carrying, we also ran models with an interaction of behaviour (with or without a visible pollen load) for both species. All models were fitted with a negative binomial distribution to account for overdispersion except for the model of bumblebee exits, which was fit with a Poisson distribution; honeybee exits were modelled with a zero-inflated negative binomial distribution. All models included a random effect structure of colony nested within site crossed with date as this reflected the structure of our experimental design. If zero-inflation was detected, we ran a zero-inflated model. The typical model structure is illustrated by (1):

1 $$\begin{aligned} activity_{i} & \sim \,distribution(\lambda_{i} ) \\ \log (\lambda_{i} ) & = \alpha_{j[i],k[i],l[i]} \, + \,\beta_{1} (temperature)\, + \,\beta_{2} (relativehumidity)\, \\ & \quad + \beta_{3} (solarradiation)\, + \,\beta_{4} (factor(wind)_{1} )\,\, + \,\beta_{5} (factor(wind)_{2} )\, \\ & \quad + \beta_{6} (factor(wind)_{3} )\, + \,\beta_{7} (factor(wind)_{4} )\, + \,\beta_{8} (factor(wind)_{5} )\, \\ & \quad + \beta_{9} (factor(wind)_{6} )\, + \,\beta_{10} (relativehumidity\, \times \,temperature)\, + \,\beta_{11} (solarradiation\, \times \,temperature)\, \\ & \quad + \beta_{12} (relativehumidity\, \times \,solarradiation) \\ \alpha_{j} & \sim N\,\left( {\mu_{\alpha j} ,\sigma_{\alpha j}^{2} } \right)\,,\quad {\text{for}}\,{\text{date}}\,{\text{j}} = 1, \ldots ,{\text{J}}\, \\ \alpha_{k} & \sim N\,\left( {\mu_{\alpha k} ,\sigma_{\alpha k}^{2} } \right)\,,\quad {\text{for}}\,{\text{site}}:{\text{colony}}\,{\text{k}} = \,1, \ldots ,{\text{K}} \\ \alpha_{l} & \sim N\,\left( {\mu_{\alpha l} ,\sigma_{\alpha l}^{2} } \right)\,,\quad {\text{for}}\,{\text{site}}\,{\text{l}} = \,1, \ldots ,{\text{L}} \\ \end{aligned}$$

where

- *i* are observations
- activity specifies bees returning, returning with pollen, or exiting the colony
- distribution is the selected model distribution
- *λ*_*i*_ is the response
- *αj[i],…,αl[i]* are the fixed-effect regressors for observation *i* grouped by the random effects:
  o *j* = *1,…,J* (date)
  p *k* = *1,…,K* (colony nested within site)
  q *l* = *1,…,L* (site)
- *β*_*1*_,…,*β*_*12*_ are the fixed-effect coefficients, which are the same for all groups
- *α*_*j*_ ~ *N(μ*_*αj*_*, σ*^*2*^_*αj*_*),…,α*_*j*_ ~ *N(μ*_*αl*_*, σ*^*2*^_*αl*_*)* indicate that the variable *α* is distributed according to the normal distribution *N* with mean vector *μ* and standard deviation *σ*^2^ for each group

We validated models using the DHARMa package (Hartig 2022). Although non-significant covariates may have had no detectable effect at our critical significance level, we retained them as we had a priori reasons for including each term. Our aim was only to test the effect of each covariate in the full model; we made enough observations to analyse the full model without the need for model selection. We tested the hypotheses of the full models of activity by comparing them with their respective null counterparts to determine if weather was predictive of bee activity (Table 2). We then calculated the Wald *χ*^2^ statistic for each covariate by removing each term from the full model to identify which covariates most influenced bee activity (Table 3). To determine if weather influenced foraging behaviour, or whether the rate of returning pollen foragers in each species changed, we modelled all returning bees per species with an interaction of behaviour (with or without pollen load) and tested the covariates using Wald’s tests as above (Table S3).

## Results

### The weather gradient

We made 458 5-min observations of both honeybee and bumblebee activity in a variety of weather conditions across the range of selected sites (Table 1).

**Table 1:** The median and range [in brackets] of each recorded weather variable at each of the seven sites. Each value is the average of that variable over a 5-min observation period, except for wind, which was only recorded once prior to each observation

Site	Temperature (°C)	Solar radiation (W m^−2^)	Relative humidity (%)	Wind (Beaufort scale)
1	13.8 [11.7–17.1]	235.5 [32.6–819.4]	63.4 [43.8–79.9]	3 [1–5]
2	14.2 [11.4–20.4]	325.0 [140.5–859.4]	60.8 [52.4–88.9]	2 [1–4]
3	15.0 [9.6–19.5]	342.3 [71.3–1041.0]	67.5 [39.2–93.2]	3 [1–6]
4	10.6 [7.8–15.1]	197.6 [70.5–803.8]	80.1 [64.1–88.1]	3 [2–5]
5	12.2 [7.4–17.9]	354.8 [29.9–981.5]	70.0 [57.1–93.5]	3 [1–5]
6	15.1 [10.9–17.6]	318.5 [89.0–885.1]	70.2 [42.3–80.5]	3 [0–4]
7	13.7 [8.7–16.8]	170.9 [66.5–887.6]	71.0 [57.2–78.7]	4 [1–5]

### Honeybee activity as a function of weather

We found that honeybees exiting the colony, returning to the colony, and returning to the colony with pollen were all explained by the recorded weather variables (Table 2a).

**Table 2:** Hypothesis test results between the full and null models of weather for each (a) honeybee and (b) bumblebee activity type observed

Activity	*n*	*χ*^2^	df	*p* value
(a) Honeybee				
Exit	448	160.34	13	< 0.001
Return	448	111.06	13	< 0.001
Return with pollen	448	140.95	13	< 0.001
(b) Bumblebee				
Exit	458	19.76	14	0.14
Return	458	17.56	14	0.23
Return with pollen	458	39.69	14	< 0.001

*χ*^*2*^ is the test statistic, *n* the number of observations, *df* the degrees of freedom, and *p* value the result of the hypothesis test. Note the difference in *n* between the two species is due to some honeybee observations being removed as the bees were swarming or the video was unusable

**Fig. 2:**
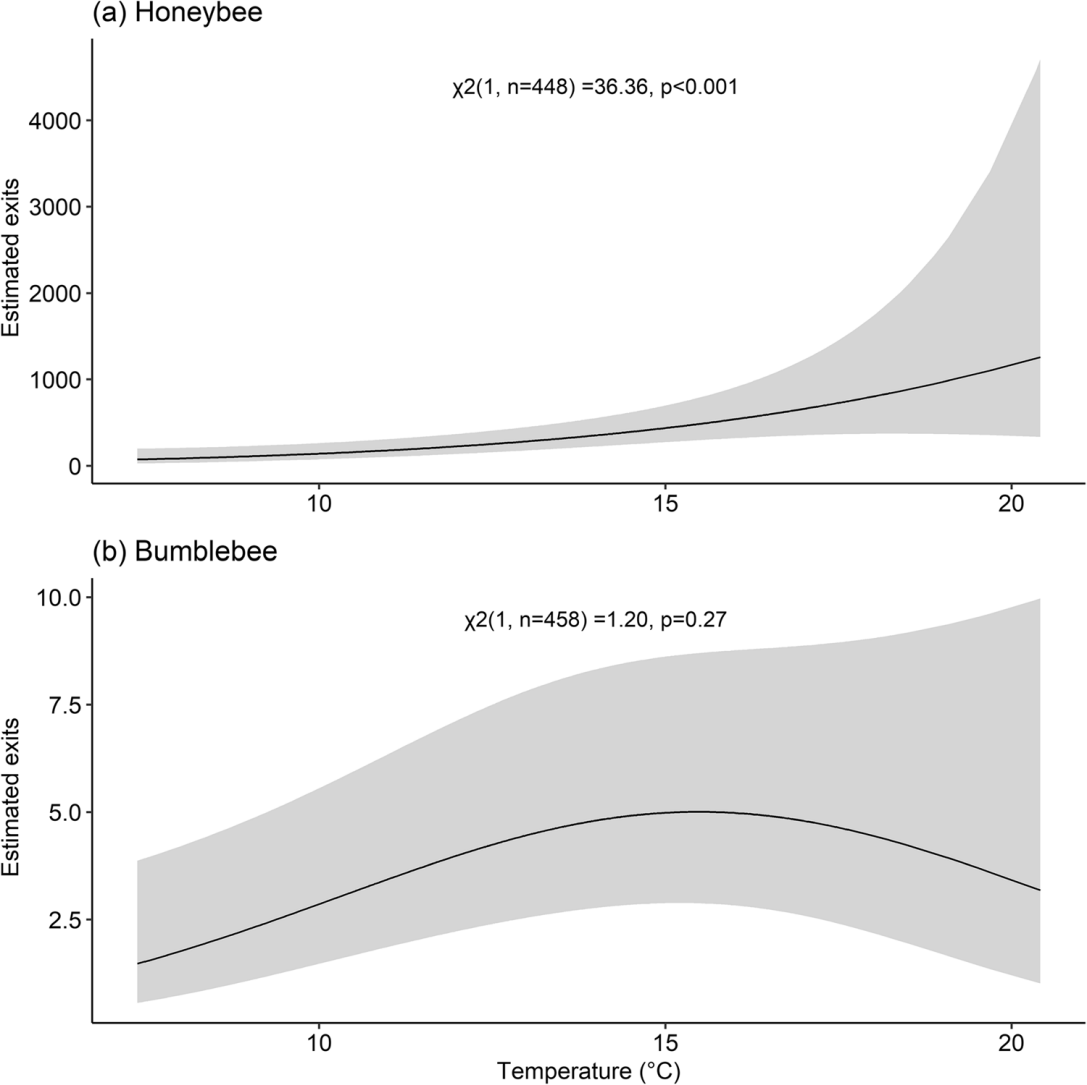
Estimates of honeybee and bumblebee exits as a function of the marginal effect of temperature and based on the models of activity. Note that we could not reject the null hypothesis that weather did not influence bumblebee exit activity. Plots are annotated with the Wald’s *χ*^2^ test statistic calculated by comparing a model without the covariate to the full model. Shaded areas indicate 95% confidence intervals for the regression lines

Honeybee exits from the colony were most related to the linear term of temperature, the linear and quadratic terms of solar radiation, and wind (Table 3a). By plotting the marginal effect of temperature (Fig. 2a), we found the number of exiting bees was predicted to rise from approximately 73 (95% CI [27, 198]) at 7 °C to ~ 1258 (95% CI [336, 4710]) individuals at 20 °C. Honeybees leaving the hive were projected to increase as solar radiation rose, this number peaked at ~ 322 individuals exiting at 417 W m^−2^ before decreasing as solar radiation rose towards 1000 W m^−2^. Honeybee exits increased until Beaufort 2 wind where it peaked, with ~ 442 individuals (95% CI [289, 676]), before declining as wind speed rose.

**Table 3:** Wald’s *χ*^2^ test statistics for the covariates in the models of (a) honeybee and (b) bumblebee activity calculated by removing each term and comparing them with the respective full models

Covariate	Returning			Returning with pollen			Exiting		
	χ^2^	*df*	*p* value	χ^2^	df	*p* value	χ^2^	df	*p* value
(a) Honeybee									
Intercept	788.69	1	< 0.001	283.73	1	< 0.001	616.66	1	< 0.001
Temp	31.96	1	< 0.001	16.93	1	< 0.001	36.36	1	< 0.001
Temp.^2^	0.001	1	0.97	0.16	1	0.69	0.10	1	0.75
RH	1.09	1	0.29	40.49	1	< 0.001	2.40	1	0.12
RH^2^	0.012	1	0.91	2.36	1	0.12	0.02	1	0.89
Solar	0.0008	1	0.96	1.32	1	0.25	8.82	1	0.003
Solar^2^	13.83	1	< 0.001	6.38	1	0.010	8.23	1	0.004
Temp.* RH	2.63	1	0.10	7.41	1	0.010	0.05	1	0.81
Solar*Temp	3.68	1	0.056	0.21	1	0.65	3.39	1	0.063
Solar*RH	19.12	1	< 0.001	5.68	1	0.020	2.68	1	0.10
Wind	19.95	4	< 0.001	7.16	4	0.21	65.58	4	< 0.001
(b) Bumblebee									
Intercept	17.44	1	< 0.001	0.53	1	0.47	29.37	1	< 0.001
Temp	0.0036	1	0.95	2.52	1	0.11	1.20	1	0.27
Temp.^2^	7.83	1	0.005	0.84	1	0.36	3.99	1	0.046
RH	0.24	1	0.62	13.30	1	< 0.001	0.30	1	0.58
RH^2^	1.22	1	0.27	6.41	1	0.011	1.38	1	0.24
Solar	3.53	1	0.06	1.44	1	0.25	0.00	1	0.99
Solar^2^	3.10	1	0.078	0.79	1	0.37	0.32	1	0.57
Temp.* RH	1.67	1	0.20	0.52	1	0.47	0.13	1	0.72
Solar*Temp	3.35	1	0.067	1.55	1	0.21	2.54	1	0.11
Solar*RH	2.80	1	0.094	1.64	1	0.20	0.59	1	0.44
Wind	2.51	5	0.78	6.58	5	0.36	1.85	5	0.87

*Temp.* is temperature (°C), *RH* is relative humidity (%), *Solar* is irradiance (W m^−2^), and *Wind* refers to wind measured on the Beaufort scale. *χ*^2^ is the test statistic, *df* the degrees of freedom, and the* p* value calculated from the hypothesis test. Shaded cells indicate a covariate below the critical significance threshold (*p* < 0.05). We included hypothesis testing of the covariates for bumblebees returning and exiting even though we could not reject the null hypotheses that weather did not influence these activities

The number of honeybees returning to the colony without pollen was most associated with the linear effect of temperature, the linear term of solar radiation, the interaction of solar radiation and relative humidity, and wind speed (Table 3a). Honeybee returns were found to be positively associated with temperature, going from ~ 60 (95% CI [24, 150]) bees at 7 °C to ~ 1187 (95% CI [377, 3742]) returning at 20 °C. As solar radiation increased, it was estimated that the number of honeybees returning to the colony would decrease from ~ 271 (95% CI [175, 420]) individuals at 30 W m^−2^ to ~ 40 (95% CI [17, 96]) bees at 1019 W m^−2^; at all measures of solar radiation more bees returned to the hive when relative humidity was lower. Honeybee returns also increased until wind speed at Beaufort scale 2, with ~ 334 individuals (95% CI [232, 482]), and decreased after this point.

**Fig. 3:**
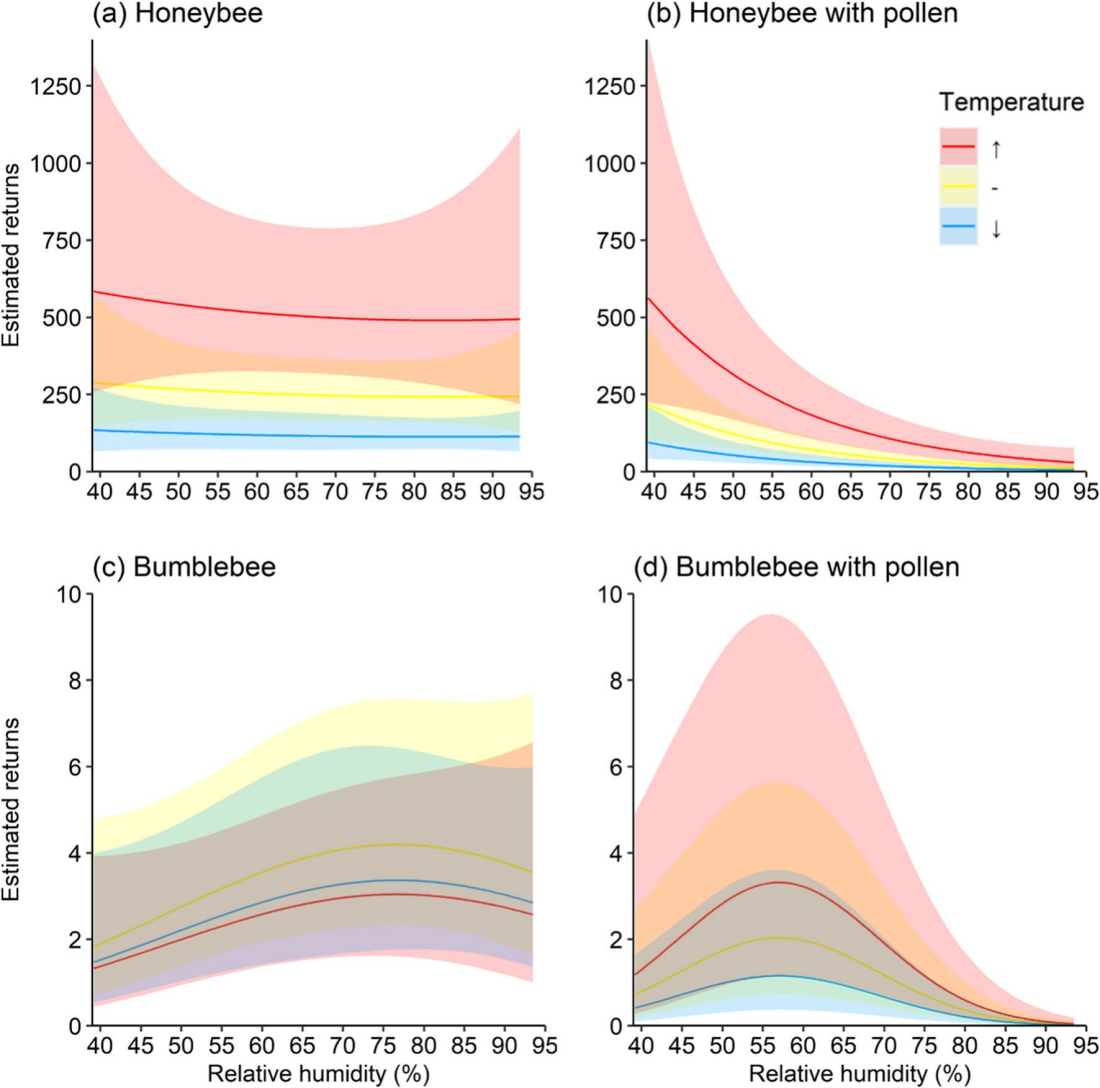
Estimates of numbers of honeybees and bumblebees returning to the colony as a function of the covariate relative humidity with an interaction of temperature and separated by behaviour (with or without a visible pollen load) based on the model. The interaction of temperature is illustrated at three levels: “↑” denotes a temperature higher than the mean, “-” represents the mean temperature recorded, and “↓” is a temperature value lower than the mean. Note that “↑” and “↓” values are equally distant from the mean. The shaded areas indicate 95% confidence intervals for the regression lines

The number of honeybees returning with pollen was related to the interactions of temperature and relative humidity, and solar radiation and relative humidity. Bees with pollen were predicted to decrease as relative humidity rose from ~ 145 bees (95% CI [54, 392]) at 39% RH to ~ 6 bees (95% CI [2, 17]) at 93% RH. This relationship was expected to change with temperature and solar radiation; at all recorded relative humidity values the number of bees returning to the colony was greater at higher temperatures (Fig. 3a), and lower at elevated solar radiation.

The interaction of relative humidity and temperature was associated with honeybee returns to the colony, with the rate bees changing depending on if individuals were carrying pollen or not (*χ*^2^(1, *n* = 448) = 8.72, *p* = 0.003). The number of honeybees collecting pollen decreased immediately as relative humidity rose, while the effect of temperature on both types of honeybee returns was expected to be strong; honeybees were more active at higher temperatures in most recorded humidities (Fig. 3a, b).

### Bumblebee activity as a function of weather

Weather did not have a significant influence on bumblebee exiting or returning to the colony when not carrying a pollen load (Table 2b; Fig. 2b). Only bumblebees returning with a pollen load were explained by weather (Table 2b), with the linear and quadratic terms of relative humidity related to the number of bees (Table 3b). We estimated a slight increase in bumblebee pollen collection until approximately 57% relative humidity before this activity decreased as humidity continued to rise.

The interaction of relative humidity and temperature was associated with bumblebee *χ*^2^(1, *n* = 458) = 7.99, *p* = 0.005) returns to the colony; the rate of bees returning varied by whether individuals carried pollen or not (Table S3). Bumblebees returning with pollen were predicted to increase until 57% relative humidity before decreasing, and more bumblebees were expected to be recorded at higher temperatures. The number of bumblebees returning without pollen loads did not vary widely with temperature or relative humidity (Fig. 3c, d).

## Discussion

### General results

All types of honeybee activity were explained by the recorded weather conditions while only bumblebee pollen collection was related to the weather. Our predictions substantiate other findings which show honeybees become more active as temperature increases and reduce flight activity with rising relative humidity and wind speeds (Benedek 1976; Vicens and Bosch 2000; Gebremedhn et al. 2014; Simioni et al. 2015). Overall, the observed conditions may have favoured bumblebee activity, as this species is able to forage at lower temperatures and lower light conditions than honeybees (Lee et al. 2016) and was less affected by weather extremes of the recorded gradient. Each type of honeybee activity varied by a wider variety of weather variables than bumblebee activity, a finding echoed in other studies (Lee et al. 2016; Nielsen et al. 2017) which suggests honeybees are more sensitive to changes in weather conditions. This indicates honeybee activity may be more strongly affected by future within-day weather conditions as the global climate changes.

### Temperature

Temperature has been observed to be the most important predictor of honeybee activity (Simioni et al. 2015; Clarke and Robert 2018). Our study was not concerned with identifying the most important weather variable as such, but we did find increasing temperature associated with an increase in all types of honeybee activity. Honeybees will begin to overheat and decrease activity above ~ 42 °C (Atmowidjojo et al. 1997), but the highest temperature we observed was ~ 20 °C. As seen elsewhere, bumblebee activity was little affected by changes in temperature (Lee et al. 2016; Nielsen et al. 2017), though this could again be a limitation of the temperatures observed as temperatures over ~ 27 °C are associated with negative effects on this species (Kenna et al. 2021); if higher temperature conditions were recorded, we might have detected a stronger effect of temperature on bumblebees.

### Solar radiation

Solar radiation and air temperature are positively related, with the former driving the latter (Peixoto and Oort 1992). Though we observed honeybee activity to be positively related to temperature, those bees decreased their activity with rising solar radiation. A solar radiation measure of 1000 W/m^2^, or the maximum normal surface irradiance, is typical for a clear day at solar noon, or when the sun is at its zenith, and reductions in honeybee activity have been observed at this time (Burrill and Dietz 1981). This could be related to the honeybee’s *zeitgedächtnis*, or ‘time sense,’ in which a bee will adhere to a diurnal pattern of activity driven by factors such as memory or the timing of reward availability (Bennett and Renner 1963; Moore and Rankin 1983; Lehmann et al. 2011).

Besides acting as a heat source, the light of the sun is necessary for bee navigation (Reber et al. 2015). Eye size allometry is established in both honeybees and bumblebees (Streinzer et al. 2013; Taylor et al. 2019): the larger eyes of bumblebees allow them to see in lower light conditions, which could explain the larger effect of solar radiation on honeybee activity and the lack of an effect on bumblebees. Reduced honeybee activity observed at lower solar radiation could be due to low sunlight in the morning or reduced visibility in cloudy conditions. This, and a decline in activity as solar radiation levels increased towards solar noon, could explain the modal pattern of honeybee activity observed and the negative effect of sunlight detected in the model interactions.

### Relative humidity

Honeybee and bumblebee foraging objective were negatively influenced by changing relative humidity; the rate of bees returning to the colony with and without pollen increased with temperature, but this was counteracted by increases in relative humidity. A visible pollen load can act as an indicator of foraging objective (Thorp 2000), and therefore, if a bee is in contact with the reproductive parts of a flower and for how long, both consequential for successful pollination (Benedek 1976; Bosch and Blas 1994). The number of honeybees returning with visible pollen loads and those without were roughly equal at the lowest relative humidity recordings, around 40%. When relative humidity increased, the number of honeybee pollen foragers decreased immediately. On the other hand, bumblebees gathered pollen at an equal rate to those without it until relative humidity reached 57%. Peat and Goulson (2005) also found relative humidity to negatively affect pollen-foraging in *B. terrestris*. They reasoned that pollen is harder to collect at higher humidities because these conditions are associated with dew and rainfall, or when there are droplets of water on the flower or the bee itself. The discrepancy in pollen-collecting between honeybees and bumblebees suggests morphological characteristics unique to each species are determinative of foraging ability (Willmer et al. 1994).

Relative humidity was also found to lower the number of honeybees returning without pollen, or likely foraging for nectar, whereas there was no effect of weather on bumblebee returns. Higher humidity can trigger nectar secretion in flowers and bumblebees may increase foraging for access to this reward despite a less-than-optimal environment (Willmer 1983); the attraction of the nectar reward could counteract any negative effect we might have expected from relative humidity, resulting in a lack of influence on this activity type.

### Wind

Wind was only found to affect honeybee return and exit activity; in both cases, the number of honeybees decreased after wind speed exceeded Beaufort scale 2, which is approximately 9 km h^−1^, or 2.5 m s^−1^. Hennessy et al. (2020) found increasing wind speed from 0 to 3 m s^−1^ increased honeybees’ reluctance to initiate flight, and this may account for the lower number of exits we observed, as well as the fewer returning, as bees may not have taken off in the first place.

There was no detected effect of wind speed on any bumblebee activity, corroborating results from Crall et al. (2017), who observed no perturbance in *B. impatiens* activity from 0.22 to 3.06 m s^−1^. Interestingly, Mountcastle et al. (2015) found *B. terrestris* with pollen loads exhibited higher flight stability and median flight speed than those without and suggest bees will forage for pollen as a means of stabilising themselves in higher winds. While we could not find a similar study of honeybees, this might explain the absence of an effect of wind (within the range recorded in this study) on pollen collection in both species.

### Pollination services in the current climate

In our study, it appears the activity of bumblebees in less sunny, windier, and more humid conditions may compensate for low honeybee activity at such times, a phenomenon known as functional complementarity (Boyle-Makowski and Philogène 1985; Kühsel and Blüthgen 2015; Lee et al. 2016). If we assume that a subset of all bees returning to and leaving a colony are foraging for floral resources, and, therefore, pollinating flowers (Corbet et al. 1993), our results illustrate how bee diversity can be important for ensuring pollination services in contemporary variable weather conditions.

We only observed two bee species, partially due to the manageability of commercial honeybee and bumblebee colonies. Honeybees of the species *Apis mellifera* are widely used in domestic agriculture the world over, and our results may be relevant for this species across its range. Bumblebees are also widely distributed and can be morphologically similar across species, so we might assume generally comparable patterns in bees of this genus; for example, thoracic temperatures between temperate and arctic bumblebees have already been found to be similar (Heinrich and Vogt 1993), although further research on less studied species is needed. Other unmanaged insect pollinators, such as solitary bees and hoverflies, are known to differentially visit flowers or deposit pollen in other weather conditions than honeybees and bumblebees (Bosch and Blas 1994). Therefore, it is likely that a higher diversity of pollinating insects could then provide a further buffer against changes in weather conditions and fortify pollination services (Brittain et al. 2013).

Many insect-pollinated crops such as apple flower early in the summer, during which weather in climates such as Ireland’s can be more varied (Met Éireann 2012; Ramírez and Davenport 2013). The early summer season is also an important time for pollinators to use floral resources while establishing colonies. Therefore, we might expect pollinator foraging objective and activity to change later in the summer, as weather conditions change or as colonies are better formed and preparing to overwinter (Kitaoka and Nieh 2009; Döke et al. 2015), and this could cause changes in the impacts of weather measured here.

### Pollination services in a changing climate

In the future, wild pollinator diversity is expected to decline with climate change (Kammerer et al. 2021) as global annual temperatures rise and within-day weather conditions change accordingly (Collins et al. 2013). In Ireland, within-day temperatures (O'Sullivan et al. 2016), the number of warm days, and heat waves are projected to increase while summer and spring rainfall will be reduced, while fewer, but more concentrated and longer-lasting precipitation events are expected in the autumn and winter (Nolan et al. 2017). By mid-century, 10-m wind speeds are predicted to decrease in all seasons, while relative humidity is expected to decrease in the south-east and increase in the north-west in the summer months (Nolan and Flanagan 2020). Our results suggest these changes may have implications for future honeybee and bumblebee activity.

Honeybee activity was positively related to temperature, and as the warmest 5% of daily maximum temperatures in Ireland are projected to increase 1.0–2.2 °C by mid-century (Nolan and Flanagan 2020), we could expect a proportional increase in activity by this species. Honeybees will begin to overheat and decrease activity above 40 °C (Atmowidjojo et al. 1997), which is not likely to be reached frequently in Ireland under current climate change predictions. However, bumblebees can overheat and decrease activity in conditions exceeding ~ 27 °C (Kenna et al. 2021). Although the projected rise in daily Irish temperatures does not reach this on average, this does not preclude extreme events in which daily temperatures could exceed the thermal limit of bumblebees and reduce their activity.

Although future decreasing wind speed in Ireland could favour honeybee and bumblebee flights, both species may forage for more pollen to stabilise themselves in higher winds regardless. Reduced precipitation in the summer could also favour bee flight and make pollen easier to gather (Lawson and Rands 2019), but a lack of plant hydration could decrease the quality of this floral reward (Corbet et al. 1979; Willmer 1983; Shrestha et al. 2018).

Although climate change may impact bee activity, it could also have a direct effect on plants and the resources they provide. Experimental warming of 1.5 °C (within the projected daily maximum increase for Ireland) has already demonstrated reductions in floral abundance, nectar volumes, and the abundance of flower-visiting insects (Moss and Evans 2022). Apple flowers are generalist, inviting a variety of insect pollinators to visit (Ramírez and Davenport 2013), but open flower morphology makes floral rewards sensitive to changes in weather. For example, nectar will more readily evaporate in warmer weather and pollen is less easily gatherable with rising humidity, both of which have implications for pollinator foraging behaviour (Corbet et al. 1979; Peat and Goulson 2005; Blažytė-Čereškienė et al. 2010). At the same time, warm and dry weather favour anther dehiscence, and more pollen could become available (Peat and Goulson 2005), though whether bees will seek it out depends on colony need (Ghosh et al. 2020). The floral rewards of crops with flowers of closed morphology or those which require manipulation could be shielded from and more resistant to such changes in the weather (Takkis et al. 2015) which influence the pattern of activity for pollinating insects (Butler 1945; Corbet et al. 1979; Willmer 1983).

## Conclusions

In the contemporary environment, bumblebee activity complements a relative lack of honeybee activity at lower temperatures and light conditions, as well as in higher humidities and wind speeds. As within-day temperatures increase and wind and precipitation decrease in the summers under Irish climate change scenarios, we expect the rates of honeybee activity to rise overall. Fluctuating weather, on the other hand, could still favour the resilience of bumblebees. Bumblebees are probably not at risk of overheating in the projected Irish weather of the next century, though they may experience negative effects during heat waves or days that exceed the projected average of daily temperatures. While we could expect pollinator activity and pollination services to improve under future conditions, this does not account for the plant response, which may produce floral rewards of lower quality in due to heat stress or a lack of precipitation, though this can vary by taxon. A diversity of pollinators with different responses to weather, and a variety of floral resources, can diminish the effect of changing weather, both now and in the future, to ensure a sustained delivery of pollination services to crops and wild plants.

## Supplementary Information

Below is the link to the electronic supplementary material.

Supplementary file1 (DOCX 28 KB)

## Data Availability

The datasets used for this study are available from the corresponding author on reasonable request.

## Code availability

The code used to generate the results are available from the corresponding author on reasonable request.
