# Supplementary material for: Responses in honeybee and bumblebee activity to changes in weather conditions
Source: Oecologia. 2023 Feb 15;201(3):689–701. doi: 10.1007/s00442-023-05332-x (PMC10038957; doi:10.1007/s00442-023-05332-x)
Supplement: Supplementary file 1 — Supplementary file1 (DOCX 28 KB) [file 442_2023_5332_MOESM1_ESM.docx]

**Responses in honeybee and bumblebee activity to changes in weather conditions**

Arrian Karbassioon^1,2^, Jon Yearlsey^2,3^, Tara Dirilgen^1,2^, Simon Hodge^1,2,4^, Jane C. Stout^4^, Dara A. Stanley^1,2^

^1^School of Agriculture & Food Science, University College Dublin, Belfield, Dublin 4, Ireland

^2^UCD Earth Institute, University College Dublin, Dublin, Ireland

^3^School of Biology and Environmental Science, University College Dublin, Dublin, Ireland

^4^School of Natural Sciences, Trinity College Dublin, Dublin, Ireland

[arrian.karbassioon@ucdconnect.ie](mailto:arrian.karbassioon@ucdconnect.ie), [dara.stanley@ucd.ie](mailto:dara.stanley@ucd.ie)

**Table S1** Details of observation sites. Sites 4,5, and 7 are in a region which historically experiences more inclement weather than the region in which 1,2,3 and 6 are situated. The distance between the northernmost (Site 7) and southernmost (Site 3) sites was 202km.

| **Site** | **Variety** | **County** | **Area (ha)** | **Flowering started** | **Flowering ended** |  |
| --- | --- | --- | --- | --- | --- | --- |
| 01 | Jonagored | Tipperary | 3.5 | 06/04/19 | 28/05/19 |  |
| 02 | Jonagored | Kilkenny | 2.8 | 06/04/19 | 28/05/19 |  |
| 03 | Cox | Waterford | 4.0 | 06/04/19 | 29/05/19 |  |
| 04 | Bramley | Dublin | 2.4 | 06/04/19 | 26/05/19 |  |
| 05 | Discovery | Dublin | 0.4 | 10/04/19 | 31/05/19 |  |
| 06 | Cox | Kilkenny | 0.5 | 21/04/19 | 29/05/19 |  |
| 07 | Mixed | Meath | 4.0 | 06/04/19 | 26/05/19 |  |

**Table S2** Table of pseudo–Variance Inflation Factors (pVIFs) for each linear and quadratic term in the models of honeybee and bumblebee activity. A pseudo-VIF for a term in a model is the ratio of squared standard errors for the term’s fitted coefficient. It is calculated by dividing the standard error of the term’s fitted coefficient from a model with only the one term and then squaring the result. Pseudo-VIFs indicate the inflation in the squared standard error of model coefficients due to collinearity. These calculations were made in the spirit of the definition of VIFs found in James *et al.* (2021)

| **Model** | **pVIF**  **Temp.**  **linear** | **pVIF**  **Temp.**  **quadratic** | **pVIF**  **RH**  **linear** | **pVIF**  **RH**  **quadratic** | **pVIF**  **Solar**  **linear** | **pVIF**  **Solar**  **quadratic** |
| --- | --- | --- | --- | --- | --- | --- |
| HB out | 2.59 | 3.30 | 1.63 | 1.93 | 1.04 | 1.28 |
| HB in pollen | 2.26 | 3.03 | 1.55 | 1.99 | 1.29 | 1.16 |
| HB in | 2.74 | 3.67 | 1.62 | 1.99 | 1.14 | 1.31 |
| BB out | 2.15 | 3.65 | 1.79 | 2.24 | 1.36 | 1.65 |
| BB in pollen | 1.78 | 2.97 | 1.64 | 1.98 | 1.42 | 1.61 |
| BB in | 2.24 | 1.69 | 1.69 | 2.13 | 1.36 | 1.71 |

**Citation:** James, G., Witten, D., Hastie, T., & Tibshirani, R. (2021). *An Introduction to Statistical Learning* (2nd ed.), pp. 101-102. New York: Springer.

**Table S3** Wald’s χ^2^ test statistics for the covariates in the models of honeybee and bumblebee behaviour (returning with or without a pollen load) calculated by removing each term and comparing them with the respective full models. *Behaviour* refers to whether a bee was observed with a pollen load or not. *Temp.* is temperature (°C), *RH* is relative humidity (%), *Solar* is irradiance (W m^-2^), and *Wind* refers to wind measured on the Beaufort scale. χ^2^is the test statistic, *df* the degrees of freedom, and the *p value* calculated from the hypothesis test. Shaded cells indicate a covariate below the critical significance threshold (p<0.05)

| **Covariate** | **Honeybee** | | | **Bumblebee** | | |
| --- | --- | --- | --- | --- | --- | --- |
|  | χ^2^ | *df* | *p value* | χ^2^ | *df* | *p value* |
| Temp. | 43.04 | 1 | <0.001 | 0.26 | 1 | 0.61 |
| Temp.^2^ | 0.37 | 1 | 0.54 | 10.84 | 1 | <0.001 |
| RH | 1.93 | 1 | 0.16 | 1.87 | 1 | 0.17 |
| RH^2^ | 0.14 | 1 | 0.71 | 1.81 | 1 | 0.18 |
| Solar | 3.24 | 1 | 0.072 | 7.15 | 1 | 0.008 |
| Solar^2^ | 11.49 | 1 | <0.001 | 5.11 | 1 | 0.24 |
| Temp.* RH | 3.067 | 1 | 0.080 | 2.83 | 1 | 0.093 |
| Solar*Temp. | 2.35 | 1 | 0.13 | 2.46 | 1 | 0.12 |
| Solar*RH | 27.80 | 1 | <0.001 | 1.83 | 1 | 0.18 |
| Wind | 17.40 | 4 | 0.002 | 1.86 | 4 | 0.87 |
| Behaviour | 130.95 | 1 | <0.001 | 4.61 | 1 | 0.032 |
| Behaviour*Temp. | 3.68 | 1 | 0.055 | 2.47 | 1 | 0.12 |
| Behaviour*Temp.^2^ | 1.18 | 1 | 0.28 | 7.06 | 1 | 0.008 |
| Behaviour*RH | 58.54 | 1 | <0.001 | 82.89 | 1 | <0.001 |
| Behaviour*RH^2^ | 0.002 | 1 | 0.97 | 24.39 | 1 | <0.001 |
| Behaviour*Solar | 3.61 | 1 | 0.057 | 3.17 | 1 | 0.075 |
| Behaviour*Solar^2^ | 1.32 | 1 | 0.25 | 9.23 | 1 | 0.002 |
| Behaviour*Temp.* RH | 8.72 | 1 | 0.003 | 7.99 | 1 | 0.005 |
| Behaviour*Solar*Temp. | 0.005 | 1 | 0.95 | 0.56 | 1 | 0.46 |
| Behaviour*Solar*RH | 0.007 | 1 | 0.93 | 3.22 | 1 | 0.068 |
| Behaviour*Wind | 1.87 | 4 | 0.76 | 8.41 | 4 | 0.13 |
|  |  |  |  |  |  |  |
